# Androgen-Driven Fusion Genes and Chimeric Transcripts in Prostate Cancer

**DOI:** 10.3389/fcell.2021.623809

**Published:** 2021-02-09

**Authors:** Mauro Scaravilli, Sonja Koivukoski, Leena Latonen

**Affiliations:** Institute of Biomedicine, University of Eastern Finland, Kuopio, Finland

**Keywords:** androgen receptor, androgens, prostate cancer, fusion gene, fusion transcript, castration-resistant prostate cancer, *TMPRSS2:ERG*, lncRNA

## Abstract

Androgens are steroid hormones governing the male reproductive development and function. As such, androgens and the key mediator of their effects, androgen receptor (AR), have a leading role in many diseases. Prostate cancer is a major disease where AR and its transcription factor function affect a significant number of patients worldwide. While disease-related AR-driven transcriptional programs are connected to the presence and activity of the receptor itself, also novel modes of transcriptional regulation by androgens are exploited by cancer cells. One of the most intriguing and ingenious mechanisms is to bring previously unconnected genes under the control of AR. Most often this occurs through genetic rearrangements resulting in fusion genes where an androgen-regulated promoter area is combined to a protein-coding area of a previously androgen-unaffected gene. These gene fusions are distinctly frequent in prostate cancer compared to other common solid tumors, a phenomenon still requiring an explanation. Interestingly, also another mode of connecting androgen regulation to a previously unaffected gene product exists via transcriptional read-through mechanisms. Furthermore, androgen regulation of fusion genes and transcripts is not linked to only protein-coding genes. Pseudogenes and non-coding RNAs (ncRNAs), including long non-coding RNAs (lncRNAs) can also be affected by androgens and *de novo* functions produced. In this review, we discuss the prevalence, molecular mechanisms, and functional evidence for androgen-regulated prostate cancer fusion genes and transcripts. We also discuss the clinical relevance of especially the most common prostate cancer fusion gene *TMPRSS2-ERG*, as well as present open questions of prostate cancer fusions requiring further investigation.

## Introduction

Androgens are steroid hormones governing the development of male reproductive tract organs and secondary male sex characteristics, as well as functioning in the regulation of muscle mass, fat deposition, and function of steroid hormone-sensitive neurons (Werner and Holterhus, 2014). Androgens are also critical for normal physiology of the male reproductive tract organs. As such, androgens and the key mediator of their functions, androgen receptor (AR), have a leading role in several diseases such as androgen insensitivity syndrome and prostate cancer (Shukla et al., 2016).

**Figure 1:**
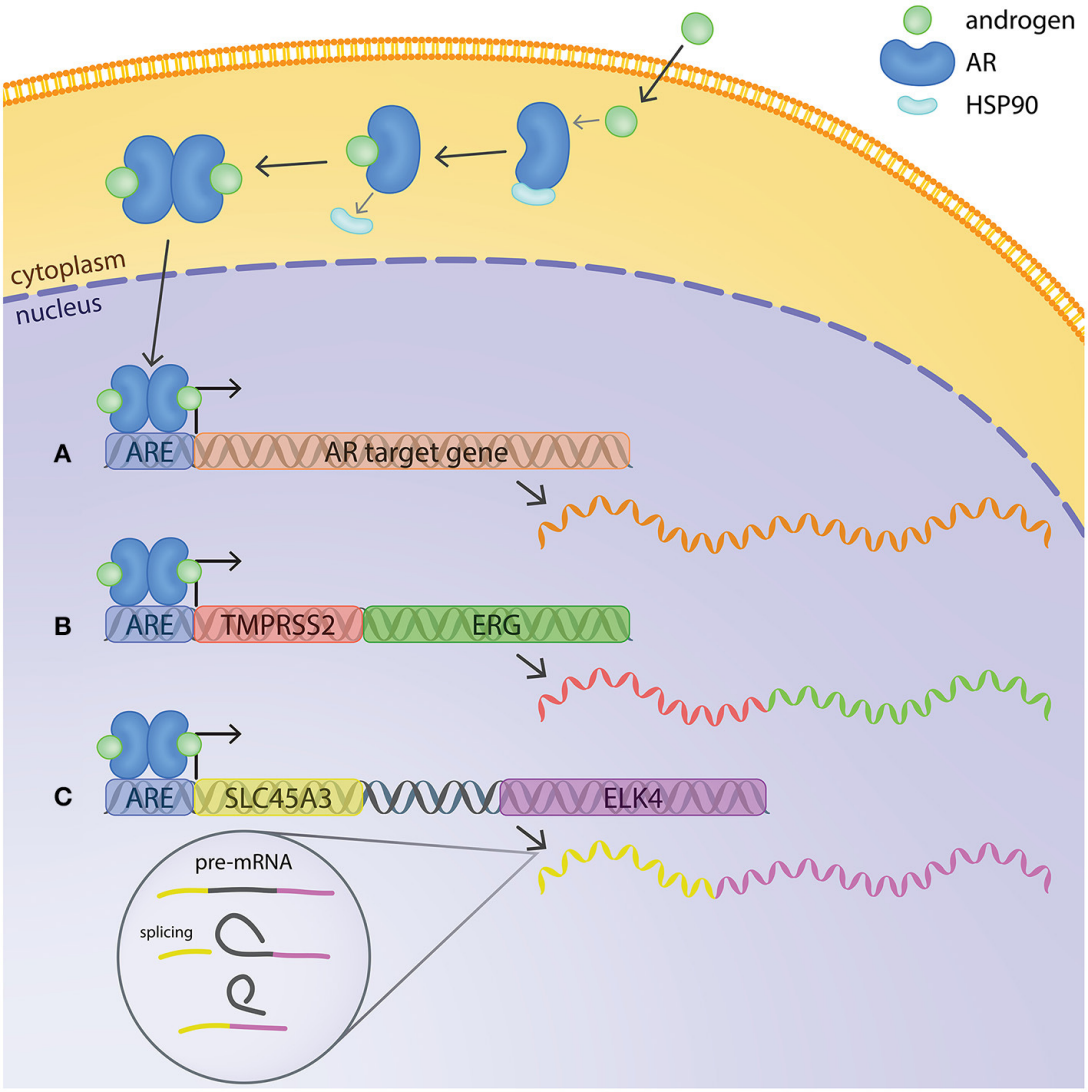
Mechanisms of androgen regulation of gene expression in prostate cancer cells. In its inactive form, AR is located in the cytoplasm bound to HSP90. The binding of androgens induces a conformational change in AR, releasing Hsp90 and enabling translocation of AR to the nucleus. AR binds to androgen response elements (AREs) at the promoter or enhancer regions of androgen-regulated genes and regulates host gene transcription. **(A)** Example of a typical androgen-regulated gene, expression of which is induced when AR binds to the ARE at the promoter region. **(B)** *TMPRSS2:ERG* fusion gene at chromosomal level and mRNA transcript. The ARE on *TMPRSS2* brings *ERG* under transcriptional regulation of AR. **(C)** *SLC45A3* and *ELK4* are located adjacently in the same chromosome. Transcription by RNA-polymerase readthrough and mRNA splicing generates SLC45A3:ELK4 fusion transcripts.

The AR is a ligand-dependent transcription factor (Figure 1). In its inhibited form, AR is located in the cytoplasm, bound to HSP90 chaperone protein. The binding of androgens (testosterone and dihydrotestosterone or DHT) induces a conformational change in AR, leading to release of HSP90 and translocation of the receptor to the nucleus. In the nuclear compartment, homodimers of AR recognize and bind to specific DNA motifs termed androgen response elements (AREs). AREs are usually located at the promoter or enhancer regions of androgen-regulated genes, and binding of AR to them usually leads to activation of host gene transcription (Figure 1) (Lamb et al., 2014). However, the regulation of target genes by AR is context-dependent, influenced by other transcriptional regulators present at the same time, leading to differences of AR transcriptional output depending on e.g., cell type and disease state (Pihlajamaa et al., 2015).

The major disease where the AR plays a key role is prostate cancer, the second most commonly diagnosed malignancy in men worldwide and the fifth leading cause of cancer-related death (Torre et al., 2015). While radical prostatectomy and/or radiation therapy represent effective treatments for primary cancer that is still confined within the prostate, there currently exists no cure for the advanced form of the disease. Advanced, metastatic prostate cancer is treated with androgen deprivation therapy (ADT), exploiting the dependence of prostate cancer cells on androgen signaling. However, most of these cases inevitably progress to castration-resistant prostate cancer (CRPC) which remains uncurable (Watson et al., 2015).

In prostate cancer, AR is responsible for the activation of specific target genes that promote cancer initiation and progression. Recent investigations revealed that AR binding to its target elements in the genome is reprogrammed during prostate tumorigenesis (Pomerantz et al., 2015). The AR also plays a crucial role in the development of castration-resistant disease (Chen et al., 2004) and the majority of CRPC cases remain dependent on AR signaling. The persistence of AR activity in the low androgen level conditions can be achieved through several AR-dependent mechanisms, including AR overexpression caused by AR gene amplification or transcriptional upregulation, AR gene mutations that increase AR activity, and expression of constitutively active AR splice variants (Coutinho et al., 2016).

**Figure 2:**
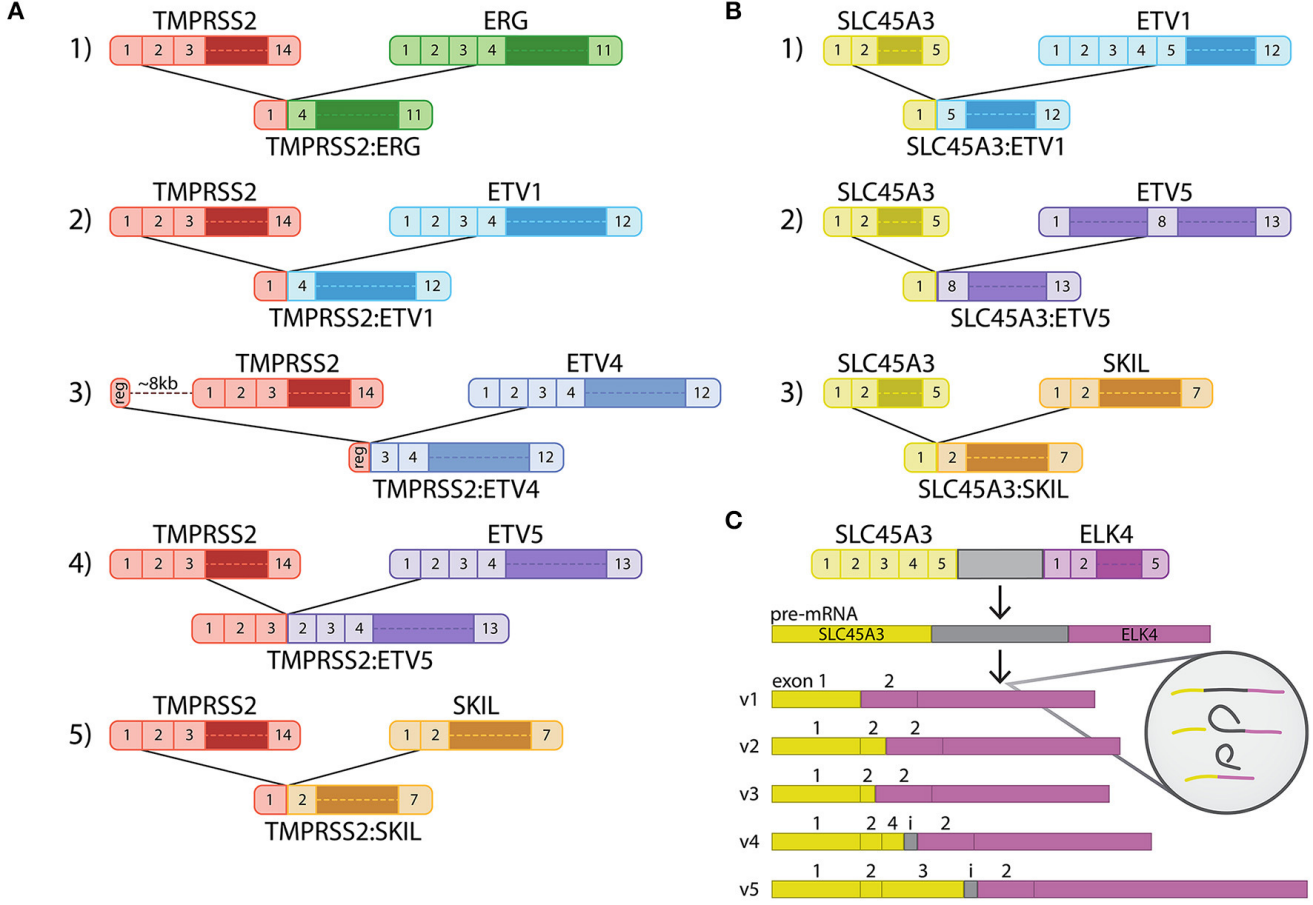
Structure of the most common and relevant androgen-driven fusion genes in prostate cancer. **(A)** Fusion genes involving *TMPRSS2* as the 5′ partner. **(B)** Fusion genes involving *SLC45A3* as the 5′ partner. **(C)** Splice variants of the chimeric SLC45A3:ELK4 fusion transcript.

At the molecular level, prostate cancer is a heterogeneous disease as revealed by recent high-throughput sequencing studies (Armenia et al., 2018). Primary prostate cancer tends to be more driven by copy number aberrations than small nucleotide variants (Fraser et al., 2017). In addition, prostate cancer commonly harbors fusion genes (Kumar-Sinha et al., 2008). Although gene fusions are found at high frequency in several rare solid cancers, many common solid cancers harbor recurrent gene fusions only at low frequencies (Kumar-Sinha et al., 2015), making prostate cancer a curious exception. In fact, the most common type of genetic alteration in prostate cancer is a structural rearrangement between an androgen-regulated gene and a member of ETS family transcription factor gene. Fusion genes of this type are found in up to ~70% of prostate cancer cases (Tomlins et al., 2005, 2006). The fusion events usually bring together an androgen-regulated 5′-part of a gene with critical 3′-protein-coding parts of the ETS genes (Figure 2). This results in androgen-induced overexpression of the ETS proteins which function as transcription factors regulating expression of genes involved in various cancer-related cellular processes, including proliferation, differentiation, transformation and apoptosis (Seth and Watson, 2005). In addition to the *ETS*-family gene fusions, several other types of fusion events also exist in prostate cancer, many of which are in a similar fashion androgen-regulated. However, not all fusions are androgen-regulated, and several 3′ fusion partners can be found fused to both androgen-regulated and androgen insensitive 5′ partners (Kumar-Sinha et al., 2015). Furthermore, not all fusion events that produce a novel androgen-regulated transcript occur at the chromosomal level. Recently, increasing evidence has revealed the presence of fusion transcripts occurring at the level of transcription and RNA, creating an interesting addition to the pool of androgen-regulated factors in prostate cancer.

## Most Common Androgen-Driven Fusion Genes in Prostate Cancer

The most prevalent genetic rearrangement in prostate cancer involves the fusion of the androgen-regulated gene *TMPRSS2* with the ETS transcription factor *ERG*, which is estimated to occur in ~50% of prostate cancer cases (Tomlins et al., 2005; Kumar-Sinha et al., 2008) being by far the single most common genetic fusion gene in solid tumors (PCAWG Transcriptome Core Group et al., 2020). In particular, more than 50% of these fusion events join the first intron of *TMPRSS2* with the third intron of *ERG* and lead to the most common *TMPRSS2:ERG* mRNA fusion transcript juxtaposing exon 1 of *TMPRSS2* with exon 4 of *ERG* (Weier et al., 2013) (Figure 2). Several other *TMPRSS2:ERG* fusion events with different junction sites have also been described to occur in prostate cancer clinical samples with lower frequency (Kumar-Sinha et al., 2008; Weier et al., 2013). The *TMPRSS2:ERG* fusion is present in the VCaP prostate cancer cell line (Tomlins et al., 2005) and has been more recently well-characterized in this model. It harbors an intragenic rearrangement between introns 1 and 4 of *TMPRSS2* and a subsequent intergenic rearrangement with intron 3 of *ERG* (Weier et al., 2013). Androgen stimulation of VCaP cells was found to cause a significant increase in ERG expression, whereas androgens did not affect ERG levels in fusion-negative LNCaP cells, confirming that the androgen regulation of *ERG* is caused by the fusion with *TMPRSS2* (Tomlins et al., 2005). Moreover, siRNA-mediated knock-down of ERG in VCaP cells significantly inhibited invasion without affecting proliferation (Tomlins et al., 2008a). Because of its prevalence and the availability of fusion-positive cell line model, the relevance of *TMPRSS2-ERG* for prostate cancer cells and the clinical manifestations of the disease has been widely studied and will be discussed in more detail in the following chapters.

*TMPRSS2* is also involved in a small percentage of rearrangements with the ETS family members *ETV1* (Tomlins et al., 2005, 2007), *ETV4* (Tomlins et al., 2006), and *ETV5* (Helgeson et al., 2008) (Figure 2). In the *TMPRSS2-ETV1* fusion event, exon 1 of *TMPRSS2* joins exon 4 of *ETV1*, resulting in a rearrangement very similar to the *TMPRSS2:ERG* fusion gene. Although LNCaP cells were reported to have a marked overexpression of ETV1 (Tomlins et al., 2005), they were not found to harbor the *TMPRSS2:ETV1* fusion (Tomlins et al., 2007). Lentiviral vector-mediated ETV1 overexpression in the immortalized prostate epithelial cell line RWPE was found to have no effect in cell proliferation, but to increase cell invasion (Tomlins et al., 2007). Moreover, androgen-mediated ETV1 upregulation in LNCaP cells was found to induce the expression of matrix metalloproteinases (MMPs) responsible for the degradation of the extracellular matrix and basal membrane. siRNA knock-down of ETV1 in androgen-dependent LNCaP cells as well as androgen-independent C81 cells was found to significantly reduce invasion and indicates a role of *ETV1* in disease progression (Cai et al., 2007). In the *TMPRSS2:ETV4* fusion event, a short regulatory region 8 Kb upstream of *TMPRSS2* and containing an androgen-regulated enhancer is juxtaposed to an intronic region immediately upstream of exon 3 of *ETV4*. This fusion gene has not been reported in prostate cancer cell lines, but native ETV4 expression is present in RWPE, PC-3 and DU145 cells and its downregulation inhibits proliferation, anchorage-independent growth and migration of prostate cancer cells (Pellecchia et al., 2012). More recently, co-expression of *ETV1* and *ETV4* was found in PC-3 and MDA-PCa-2b prostate cancer cell lines, representing models of advanced disease. Silencing of either ETS family member did not affect proliferation or apoptosis. However, *ETV4* knock-down cells presented a significant decrease in colony formation, whereas *ETV1* knock-down cells showed a significant decrease in cell invasion, confirming a relevant role of *ETV1* in disease progression (Mesquita et al., 2015). In the *TMPRSS2:ETV5* fusion event, exons 1 to 3 of *TMPRSS2* are fused to exon 2 of *ETV5*. Although this rearrangement is also absent in all known prostate cancer cell lines, functional studies of ETV5 overexpression performed on the RWPE model produced very similar results to those obtained with the RWPE-ETV1 overexpression model (Helgeson et al., 2008).

More recently, a novel fusion event was reported in about 1% of prostate cancer cases, juxtaposing exons 1 or 2 of *TMPRSS2* to exon 2 of the SMAD inhibitor and oncogenic factor *SKIL* and leading to its overexpression. Downregulation of SKIL expression in PC-3 cells was found to reduce cell growth, invasion and colony formation, whereas SKIL overexpression in RWPE cells showed a marked increase in invasive potential (Annala et al., 2015). *ETV1, ETV5*, and *SKIL* have also been found to be 3′-prime fusion partners with the prostate-specific, androgen-induced gene solute carrier family 45, member 3 (*SLC45A3*), also referred to as prostein, as a 5′ partner (Tomlins et al., 2007; Helgeson et al., 2008; Annala et al., 2015) (Figure 2). In these fusion events, exon 1 of *SLC45A3* is juxtaposed to exon 5 of *ETV1* (Tomlins et al., 2007), exon 8 of *ETV5* (Helgeson et al., 2008) or exon 2 of *SKIL* (Annala et al., 2015). These *SLC45A3-ETS* fusions and the fusions involving *SKIL* have not been reported in prostate cancer cell lines. However, a recent study has shown that concomitant treatment of LNCaP cells with androgens and irradiation induced TMPRSS2:ERG, TMPRSS2:ETV1 and SLC45A3:ETV1 transcript expression. Genomic sequencing confirmed the authenticity of the fusion events at chromosomal level, suggesting a potential role of AR in promoting tumor translocations (Lin et al., 2009).

Recently, Chakravarthi and colleagues identified a fusion occurring in around 30% of primary prostate cancer cases and involving the AR target gene *KLK4* as a 5′ partner and the non-coding pseudogene *KLKP1* (Chakravarthi et al., 2019). Both KLK4 and KLKP1 belong to the kallikrein family of serine proteases, and their genes are located adjacent to each other in a cluster of 15 genes on chromosome 19 (q13.33–q13.41), containing also the well-known *KLK3* (PSA). The KLK4-KLKP1 fusion is formed either by a trans-splicing mechanism or an in-frame fusion due to a microdeletion, leading to the fusion of the first two exons of *KLK4* with exon 4 and 5 of *KLKP1*. The resulting chimeric sequence predicts a 164–amino acid protein, of which the latter third is derived from KLKP1, leading to a conversion of the non-coding pseudogene to a protein-coding gene. Utilizing cell culture and chicken chorioallantoic membrane (CAM) assay, the expression of KLK4-KLKP1 fusion transcript was shown to affect cell proliferation, cell invasion, intravasation, and tumor formation (Chakravarthi et al., 2019).

In addition, transcriptome sequencing of ETS-fusion-negative prostate cancer revealed genetic rearrangements involving RAF-kinase family members, namely SLC45A3-BRAF and ESRP1-RAF1, recurrent in about 2% of advanced PCa cases, the former one being AR-regulated (Palanisamy et al., 2010). Ectopic expression of both chimeras in prostate epithelial cells showed an increase in oncogenic properties, and these RAF-kinase fusion genes are generally associated with features of advanced disease, such as high Gleason score and castration resistance (Palanisamy et al., 2010; Beltran et al., 2013; Ross et al., 2016; Pederzoli et al., 2020). In addition to the fusion genes mentioned above, a significant number of other prostate cancer fusion genes have been described in clinical material (Tomlins et al., 2007; Kumar-Sinha et al., 2008; Weier et al., 2013). Most of these occur with very low frequencies and/or have not been studied further, often due to lack of cell models expressing them.

## Androgen-Driven Fusion Transcripts in Prostate Cancer

Recent evidence has shown that, in addition to fusions at the genetic level, also chimeric fusion transcripts may be relevant for prostate cancer. As mentioned already above, the KLK4-KLKP1 fusion, combining sequences of a protein-coding gene and a pseudogene, may potentially be formed by a trans-splicing mechanism (Chakravarthi et al., 2019). So far, the most studied one in prostate cancer is the one where *SLC45A3* is involved in the generation of a chimeric transcript with *ELK4* (Figure 1). *ELK4* has been previously described as a growth-promoting androgen receptor target in LNCaP cells and has been shown to be overexpressed in a subset of prostate tumors (Makkonen et al., 2008). SLC45A3-ELK4 mRNA expression was later confirmed in prostate cancer samples, as well as in LNCaP cells, and five different mRNA variants of the chimeric transcript were described (Rickman et al., 2009). The most common form consists of exon 1 of SLC45A3 joined to exons 2 of ELK4, two other forms showed exon 1 and 2 of SLC45A3 joined to exon 2 of ELK4, a fourth variant includes exon 1, 2 and part of exon 4 of SLC45A3 (with a short intergenic sequence) joined to exon 2 of ELK4 and the last variant consists of exon 1–3 of SLC45A3 (including the same short intergenic sequence) fused to exon 2 of ELK4 (Rickman et al., 2009) (Figure 2). *SLC45A3* and *ELK4* are located adjacent to each other on chromosome 1 and in this case the generation of the chimeric transcript is not caused by a chromosomal rearrangement, as described for *TMPRSS2:ERG* and *SLC45A3-ETV1*, but rather by *cis*-splicing of adjacent genes/gene read-through (Zhang et al., 2012). Moreover, the SLC45A3-ELK4 transcript was shown to be induced by androgens and the chimeric mRNA, but not the wild-type ELK4, was found to drive androgen-dependent proliferation in prostate cancer cells (Zhang et al., 2012). Other examples of chimeric transcripts generated by a *cis*-splicing of adjacent genes were later described in prostate cancer samples. However, they involve genes that are not androgen regulated and are not specific to cancer but were also found in normal prostate (Qin et al., 2015, 2017), indicating that this mechanism is not unique to cancer cells.

## Connections of AR-Driven Fusion Genes and Long Non-Coding RNAs in Prostate Cancer

When a fusion gene coding for a transcription factor is present and expressed, the transcriptional program of the prostate cancer cells is affected. For example, when AR drives expression of *ERG* from the common *TMPRSS2:ERG* fusion, both of these factors have been recently described to be involved in the regulation of long non-coding RNAs (lncRNAs) in prostate cancer. LncRNAs are >200 bp long RNAs that do not encode for protein end-products. They are known to play important roles in the regulation of gene expression and to be dysregulated in several types of human malignancies, including prostate cancer (Martens-Uzunova et al., 2014).

In a recent report, the transcriptomes of primary tumors, castration-resistant prostate cancers and benign prostatic hyperplasia controls were deep-sequenced with the aim of identifying prostate cancer-specific lncRNAs associated with more advanced stages of the disease. Interestingly, the expression of a novel lncRNA (*PCAT5*) was shown to be strongly correlated with *ERG* expression in ERG-positive primary tumors, as well as CRPCs (Ylipaa et al., 2015). The expression of PCAT5 was confirmed in the VCaP prostate cancer cell line harboring the *TMPRSS2:ERG* fusion and was significantly decreased as a result of siRNA-mediated knock-down of ERG. In addition, siRNA-mediated knock-down of PCAT5 expression in ERG-positive DuCaP cells significantly reduced cell growth (Ylipaa et al., 2015). Altogether, this study revealed the role of ERG in driving the expression of a growth-promoting ncRNA. In a later investigation, it was shown that several other prostate cancer-associated lncRNAs or PCATs are correlated with *ERG* expression and are significantly down-regulated by ERG knock-down in both VCaP and DuCaP cells (Kohvakka et al., 2020). Moreover, the majority of these PCATs were found to be also regulated by the AR, and analysis of previously published ChIP data (Pomerantz et al., 2015) revealed that most of the sites bound by ERG in PCATs were co-occupied by the AR (Kohvakka et al., 2020), confirming the previous findings by Yu and colleagues who reported the co-occupancy of AR and ERG in prostate cancer cells (Yu et al., 2010). Kohvakka and colleagues further demonstrated that the ERG- and AR-regulated lncRNA EPCART (ERG-positive PC-associated androgen responsive transcript) is functionally relevant for prostate cancer, as knockout of *EPCART* reduces migration and proliferation of LNCaP cells. Moreover, high expression of EPCART was associated with biochemical recurrence in prostatectomy patients and was found to be an independent prognostic marker in primary prostate cancer (Kohvakka et al., 2020).

Fusion events other than the *TMPRSS2:ERG* are also associated with the regulation of lncRNAs in prostate cancer. As described above, the SLC45A3-ELK4 fusion transcript is generated by *cis*-splicing of adjacent genes/gene read-through, rather than by actual genomic rearrangement (Zhang et al., 2012). A later study showed that this fusion transcript functions as a long non-coding chimeric RNA (lnccRNA). SLC45A3-ELK4 lnccRNA was found to be <1% of the expression level of the native ELK4 mRNA and therefore would only contribute to a minor percentage of the total ELK4 protein pool in prostate cancer cells. Selective siRNA-mediated knock-down of the fusion transcript proved effective at reducing cell proliferation rate, whereas ELK4 mRNA knock-down had no such effect. Moreover, a mutant SLC45A3-ELK4 transcript with an early stop codon, and therefore unable to generate a functional ELK4 protein product, rescued the proliferation of siSLC45A3-ELK4 treated cells, highlighting the functional role of the chimeric RNA. SLC45A3-ELK4 mutant characterization showed that exon1 and exon3 of ELK4 are needed for the chimeric transcript to exert its rescue activity, and functional studies showed that the chimeric transcript represses the expression of *CDKN1A* and therefore promotes cell cycle progression from G1 to S phase (Qin et al., 2017).

## Molecular Functions of *TMPRSS2:ERG* Fusion Gene–the Most Common Fusion Gene in Prostate Cancer

More than 90% of prostate cancer samples that overexpress ERG harbor the *TMPRSS2:ERG* fusion gene (Tomlins et al., 2005; Demichelis et al., 2007). The first investigations on the molecular mechanisms of ERG overexpression driven by the fusion gene were performed by Tomlins and colleagues. They showed that the *TMPRSS2:ERG* positive VCaP prostate cancer cells overexpressed the fusion product when treated with synthetic androgens (Tomlins et al., 2005). Further experiments were performed on RWPE benign, immortalized prostate epithelial cells infected with a lentivirus expressing the truncated ERG product analogous to the one deriving from the *TMPRSS2:ERG* fusion gene. These cells showed increased invasion capabilities, although no changes were observed in cell proliferation. However, the overexpression of ERG was not sufficient to cause transformation of the cells (Tomlins et al., 2008a). Moreover, Tomlins and colleagues generated transgenic mice expressing the same truncated product specifically in the prostate (under a probasin promoter) and showed that about 40% of mice developed PIN lesions, with disruption of the basal cell layer, but not prostatic adenocarcinoma (Tomlins et al., 2008a). Similar findings were also reported by Klezovitch and colleagues (Klezovitch et al., 2008).

Carver and colleagues also showed that *ERG* rearrangements are often associated with loss of the tumor suppressor *PTEN*, which had also previously been reported in almost 50% of HGPIN lesions (Bettendorf et al., 2008). Pten heterozygous mice overexpressing ERG specifically in the prostate (*Pten*^+/−^*;Probasin-ERG*) developed prostatic adenocarcinoma, whereas *Pten*^+/−^ mice only showed HGPIN lesions. Moreover, two genes involved in promoting cell migration and invasion (*CXCR4* and *ADAMTS1*) were found to be upregulated in the context of ERG overexpression (Carver et al., 2009). Another study performed on xenograft models using VCaP cells with knocked-down *ERG* expression (siRNA), showed a significant reduction in tumorigenicity, concomitant reduction in the expression of the oncogene *C-MYC* and upregulation of prostate epithelial differentiation genes *KLK3* and *SLC45A3* (Sun et al., 2008), suggesting that *ERG* overexpression has an oncogenic role in established prostate tumors, by inducing upregulation of *C-MYC* and repressing prostate epithelial differentiation. Later, Yu and colleagues performed chromatin immunoprecipitation coupled with massively parallel sequencing (ChIP-Seq) in LNCaP and VCaP cells. The results revealed that ERG and AR can co-occupy the same target genes and ERG functions as a repressor of AR-driven lineage-specific differentiation program. ERG also directly regulates the expression of the histone methyltransferase *EZH2*, by binding to its promoter and activating EZH2-mediated cell de-differentiation program (Yu et al., 2010). Interestingly, the transcriptional role of ERG described by Yu and colleagues is in contrast with *ETV1* transcriptional activity in prostate cancer. A recent study demonstrated that ERG and ETV1 can regulate a common set of AR target genes, but in an opposite fashion. In particular, ERG negatively regulates the androgen receptor (AR) transcriptional program, whereas ETV1 was found to upregulate genes involved in AR signaling and cooperates in its activation.

These findings were confirmed both *in vitro* and *in vivo* and pointed to a role of the ETV1 transcriptional program in the development of more aggressive disease and poorer clinical outcome (Baena et al., 2013).

Several investigations on the role of the fusion gene have been performed using cell line models of prostate cancer and non-tumorigenic prostate epithelial cells. Klezovitch and colleagues used immortal but non-tumorigenic BPH-1 human prostate epithelial cells with overexpression of a truncated form of ERG analogous to the one derived from the most common *TMPRSS2:ERG* fusion gene (*TMPRSS2* exon 1 and *ERG* exon 4). These authors, in contrast to what reported by Tomlins and colleagues (Tomlins et al., 2008a), found that ERG overexpression increased the growth of BPH-1-ERG compared to native BPH-1 cells. Moreover, BPH-1-ERG cells also showed higher invasion rate, but no effect was observed on migration. They reported similar results using RWPE-1 cells and in both cases, the addition of plasminogen activator inhibitor (PAI-1) completely eliminated the difference in invasion rate between native and ERG overexpressing cells (Klezovitch et al., 2008). These latest findings were confirmed by Tomlins and colleagues in VCaP cells. They showed using ChIP that urokinase plasminogen activator (*PLAU*) is a direct target of ERG in VCaP cells and that PAI-1 inhibited the invasion of VCaP cells (Tomlins et al., 2008a).

Cai and colleagues showed that *ERG* and *CXCR4*, which has been previously shown to contribute to the formation of bone metastases (Chinni et al., 2008), are both overexpressed in the fusion-positive VCaP cells, compared to PC-3 cells (Cai et al., 2010). Moreover, ChIP experiments performed in VCaP revealed that ERG binds within the *CXCR4* promoter in VCaP cells. Synthetic androgen (R1881) treatment of VCaP and LNCaP cells showed increased expression of both *ERG* and *CXCR4* in VCaP, but not LNCaP, suggesting that, indeed, the androgen-mediated *ERG* overexpression caused by the *TMPRSS2:ERG* fusion drives *CXCR4* expression in VCaP cells, confirmed by the lack of *CXCR4* induction when siERG-VCaP cells were treated with R1881. Androgen-induced *CXCR4* overexpression was also shown to increase invasiveness of VCaP cells (Cai et al., 2010). These results are in accordance with the data shown earlier by Carver and colleagues (Carver et al., 2009) and altogether reveal a role of the TMPRSS2:ERG fusion in the progression to advanced disease.

More recent efforts have revealed several other downstream effectors of *ERG* in prostate cancer. Stable knock-down of *ERG* expression in VCaP cells was shown to lead to increased expression of active β1-integrin and E-cadherin, both responsible for cell adhesion (Gupta et al., 2010), supporting the previous finding of increased invasion in *ERG* overexpressing cells (Tomlins et al., 2008a) and highlighting a role of *ERG* in epithelial to mesenchymal transition (EMT). Moreover, *ERG* overexpression was shown to activate the Wnt signaling pathway via increased expression of the Wnt receptor *FZD4* (Gupta et al., 2010). Subsequently, stable populations of immortalized prostate epithelial cell lines BPH-1, PNT1B, and RWPE-1 overexpressing *ERG* were also shown to undergo EMT and acquire invasive characteristics with downregulation of cell adhesion molecules (E-cadherin) and upregulation of the EMT mediator integrin-linked kinase (ILK) and its downstream effectors Snail and LEF-1 (Becker-Santos et al., 2012). *ERG* expression driven by the *TMPRSS2:ERG* fusion was also associated with increased expression of *SOX9*, a transcription factor required for prostate development and involved in the maintenance of stem/progenitor cells. The correlation between ERG and SOX9 protein levels was verified in clinical samples of prostate cancer by IHC and in androgen-treated VCaP cells (Cai et al., 2013). Transgenic overexpression of *SOX9* in the prostate of mice caused the development of PIN, as observed previously with *ERG* overexpression by Tomlins et al. (2008a). Moreover, overexpression of *SOX9* in LNCaP cells significantly increased cell invasion and the same effect was observed in doxycycline-inducible SOX9-VCaP cells treated with siRNA for *ERG* after *SOX9* induction, suggesting that the invasive phenotype caused by *ERG* overexpression is mediated by *SOX9* activation. ChIP experiments showed that ERG binds and opens the regulatory region for an AR-regulated enhancer of *SOX9* expression (Cai et al., 2013). Another study connected *ERG* to miR-200c, a member of the miR-200 family. ERG was shown to directly repress the expression of miR-200c, by binding an ETS motif in its promoter. Decreased miR-200c expression causes reactivation of its target gene *ZEB1*, an important mediator of EMT (Kim et al., 2014).

## Clinical Implications of AR-Driven Fusion Genes in Prostate Cancer

### Diagnostic and Prognostic Implications of TMPRSS2:ERG

Due to its high frequency in prostate cancer cases, the *TMPRSS2:ERG* fusion is considered to be relevant for the disease. However, its correlation to prostate cancer development and progression, as well as its clinical significance are not yet fully understood. Since the discovery of the fusion gene and its prevalence in the disease, several studies have also been performed with the aim of assessing its potential use as a diagnostic or prognostic marker with conflicting results.

The *TMPRSS2:ERG* fusion gene is not present in non-neoplastic prostate epithelium, but has been described in high-grade prostatic intraepithelial neoplasia (HGPIN) lesions (Cerveira et al., 2006; Park et al., 2010), suggesting that it might represent an early event in the development of prostate cancer (Perner et al., 2007). Subsequently, a seminal study on the biological role of aberrant ERG expression showed that, in fact, *ERG* rearrangements are not frequently found in HGPIN. Evaluation of HGPIN lesions with adjacent adenocarcinoma revealed that few cases showed rearrangements in the lesions and when present, they were always detected in the adenocarcinoma as well. Conversely, several cases harbored rearrangements in the adenocarcinoma, but not in HGPIN lesions (Carver et al., 2009). This suggests that the *TMPRSS2:ERG* fusion gene is an early event but associated with progression from HGPIN to adenocarcinoma.

Population-based studies using watchful-waiting patient cohorts showed a significant association between the presence of the fusion gene and poorer clinical outcome, defined as development of distant metastases or cancer-related death (Demichelis et al., 2007; Attard et al., 2008). Moreover, investigations performed on retrospective cohorts of prostate cancer patients undergoing radical prostatectomy revealed that the fusion gene is associated with more advanced tumor stage (Perner et al., 2006), earlier biochemical recurrence (Yoshimoto et al., 2008) and lymph node metastases and seminal vesicle invasion (Wang et al., 2006). In contrast, other retrospective studies showed opposite findings. The fusion gene was either associated with significantly longer biochemical recurrence-free survival (Saramaki et al., 2008; Hermans et al., 2009; Boormans et al., 2011), or not significantly associated with clinical outcome (Gopalan et al., 2009; Minner et al., 2011; Toubaji et al., 2011). These findings suggest that ERG overexpression driven by the *TMPRSS2:ERG* fusion might represent a highly diagnostic marker rather than prognostic. More recently, combined detection of urinary prostate cancer gene 3 (*PCA3*) and *TMPRSS2:ERG* has been shown to improve the sensitivity for prostate cancer diagnosis (Robert et al., 2013).

Subsequent investigations have shown that the fusion gene can generate several different TMPRSS2:ERG transcripts via alternative splicing (Hu et al., 2008; Wang et al., 2008). These variants can be grouped into two types. Type I variants encode full length ERG proteins, whereas type II variants encode a shorter version of ERG, lacking the ETS domain (Hu et al., 2008). Interestingly, type II splice variants were found to be more abundantly expressed in prostate cancer clinical samples, as well as in VCaP cells (Hu et al., 2008). The relative amount of type I/type II splice variants has also been found to correlate with clinical features of prostate cancer patients. A higher ratio of type I/type II was correlated with poorer outcome (Hu et al., 2008) and type II variants can function in a dominant-negative fashion by interfering with the transcriptional regulatory function of type I variants (Rastogi et al., 2014). Moreover, a recent study showed an association between increased retention of a 72 bp exon (exon 11) in the ERG transcript and more advanced stages of the disease (Hagen et al., 2014).

### Role of TMPRSS2:ERG in Advanced and Metastatic Prostate Cancer

While the *TMPRSS2:ERG* fusion gene is an early event of prostate tumorigenesis and associated with progression from HGPIN to adenocarcinoma, the role of this genetic alteration in more advanced and metastatic disease has also been recently investigated. Already in the initial report on the identification of the *TMPRSS2:ERG* fusion gene, fusion transcripts where detected in clinical specimens of metastatic, castration-resistant prostate cancer (Tomlins et al., 2005). Later, mouse xenografts derived from primary tumors, local and distant metastases of fusion-positive prostate cancers were used to study the expression of *ERG*. All androgen-dependent, fusion-positive xenografts were shown to overexpress *ERG*, including samples derived from local and distant metastases. In contrast, AR-negative and fusion-positive xenografts, all derived from metastases, did not express *ERG*, consistently with the model of AR-driven *ERG* expression from the fusion gene. These results demonstrate that *ERG* overexpression is also present in more advanced stages of the disease in AR-positive samples, but it is bypassed in androgen-independent tumors (Hermans et al., 2006).

Interestingly, a subsequent study reported that the NCI-H660 cell line, derived from a metastatic site of an extrapulmonary small cell carcinoma arising from the prostate, harbors the *TMPRSS2:ERG* fusion (Mertz et al., 2007). NCI-H660 cells are androgen-independent, as opposed to the androgen-dependent VCaP cell line derived from the vertebral bone metastasis of a hormone-refractory prostate tumor (Korenchuk et al., 2001). Moreover, NCI-H660 cells overexpress *ERG* from the *TMPRSS2:ERG* fusion gene in an androgen-independent fashion, suggesting that the fusion gene might have a role in AR-negative tumors as well (Mertz et al., 2007). *ERG* expression was later examined in samples of fusion-positive, androgen-dependent primary prostate cancers and CRPC samples, as well as in VCaP xenografts before and after castration, with the aim of establishing whether TMPRSS2:ERG fusion transcript expression is reactivated in CRPC after androgen deprivation therapy. The results showed that ERG expression levels were comparable in samples of fusion-positive primary tumors and fusion-positive, AR-overexpressing CRPCs, suggesting that AR overexpression at least partly reactivates TMPRSS2:ERG transcript expression in CRPC samples to levels similar to those present in the primary tumors. This was confirmed in VCaP cells/xenografts showing declining levels of ERG transcripts and protein in response to removal of androgens and reactivation of ERG expression in VCaP xenografts that relapsed and showed AR reactivation (Cai et al., 2009). Attard and colleagues used circulating tumor cells (CTCs), primary prostate tumor and CRPC samples from fusion-positive prostate cancers to study the *ERG* status and expression. The results showed that the *ERG* status in CTC matched the status in tumor samples, both primary tumors and CRPC. Moreover, *ERG* expression was detected and maintained in CRPC samples as well, indicating that hormone regulation of fusion-derived *ERG* expression is retained in the more advanced stages of the disease (Attard et al., 2009).

More recent studies examined the functional role of the *TMPRSS2:ERG* fusion gene in metastatic prostate cancer. Tian and colleagues used a newly established prostate cancer cell line (PC3c), derived from PC-3, to assess the role of the TMPRSS2:ERG fusion transcript in the formation of bone metastases. They used PC3c clones that overexpress the most common TMPRSS2:ERG transcript variant (TMPRSS2 exon 1 and ERG exon 4) at variable levels and also including the 72 bp exon 11 previously shown to be associated with more advanced stages of the disease (Hagen et al., 2014). PC3c cells, like the parental PC-3 cells, are both AR- and *TMPRSS2:ERG* fusion-negative, but unlike PC-3 cells, can rapidly generate mixed bone lesions *in vivo*, whereas PC-3 cells only generate pure osteolytic bone lesions. Therefore, PC3c represent a better model of prostate cancer bone metastasis as it recapitulates the commonly observed mixed lesions found in advanced prostate cancer clinical cases (Fradet et al., 2013). The results of TMPRSS2:ERG overexpression in PC3c revealed no effect on cell proliferation compared to native PC3c, but a significant increase in both cell migration and invasion in a dose-dependent manner. Moreover, global gene expression analysis of the TMPRSS2:ERG overexpressing clones compared to native PC3c showed a significant upregulation of the genes for the metalloproteinase *MMP9* and transmembrane glycoprotein, semaphorin co-receptor Plexin-A2 (*PLXNA2*). These genes were confirmed to be directly regulated by ERG overexpression. Knock-down experiments confirmed that *PLXNA2* is directly involved in the increased migration and invasion capabilities of prostate cancer cells (Tian et al., 2014), providing insight into the molecular mechanisms of action of the fusion transcript in metastatic disease. ERG binding sites in *MMP9* were also previously shown in ChIP experiments performed in VCaP cells (Yu et al., 2010). Similar functional results were obtained by Deplus and colleagues using the highly metastatic PC-3M cell line with stable luciferase expression (PC3-M-luc) and overexpression of the TMPRSS2:ERG fusion transcript. As previously shown by Tian et al. (2014), the overexpression of the fusion transcript did not affect cell proliferation, but increased cell migration and invasion compared to native PC-3M-luc (Yoshimoto et al., 2008). Moreover, a significant increase in tumor growth was observed when the cells were subcutaneously injected in mice, as well as a significant increase in tumor dissemination with intracardiac injection mimicking the hematogenous dissemination of metastatic cells (Deplus et al., 2017). These results provide further evidence on the role of *TMPRSS2-ERG* in advanced prostate cancer and specifically in tumor cell dissemination into the bone.

A more recent study provides yet more data supporting the involvement of the *TMPRSS2:ERG* fusion gene in bone metastasis progression. The same clones of TMPRSS2:ERG overexpressing PC3c cells described by Tian et al. (2014) were used for direct injection into the tibiae of SCID mice. Compared with native PC3c, the fusion-overexpressing cells generated larger bone formation areas and smaller bone destruction areas, overall larger bone volume and reduced osteoclast surface, indicating an enhanced osteoblastic phenotype and inhibition of osteoclastic destruction *in vivo* (Delliaux et al., 2018). Overexpression of TMPRSS2:ERG was found to induce the expression of the osteoblastic markers Collagen Type I Alpha 1 Chain (*COL1A1*) and Endothelin-1 (*ET-1*), responsible for improved acquisition of a bone-like phenotype in cancer cells (osteomimicry), helping the cancer cells survive in the bone microenvironment (Delliaux et al., 2018). Altogether, the data from these latest studies reveal an important role of *ERG* in the dissemination of metastatic cells, the seeding to the bone as a preferential metastatic site and the generation of metastatic lesions in prostate cancer.

### Fusion Co-occurrence and Multifocal Nature of Prostate Cancer

Prostate cancer is a heterogeneous disease which very often harbors multiple cancer foci within the same gland. It is well-established that different foci are histologically and molecularly heterogeneous, suggesting that they are clonally independent (Wise et al., 2002; Arora et al., 2004). The study of fusion genes in the context of multifocal disease has provided significant insight into tumor clonality [recently reviewed in Pederzoli et al. (2020)]. Assessments of TMPRSS2 rearrangements by fluorescence *in situ* hybridization (FISH) in separated foci of prostate cancers revealed interfocal heterogeneity and intrafocal homogeneity, indicating that individual foci are the result of clonal expansion (Mehra et al., 2007). Similar results were shown by FISH analysis of TMPRSS2-ERG rearrangements in multifocal prostate cancers (Barry et al., 2007). TMPRSS2-ETS rearrangements were later characterized in prostate cancer metastases and different metastatic sites from the same patients were found to harbor the same molecular sub-type of gene fusion events, indicating clonal expansion of advanced disease from a single primary focus (Mehra et al., 2008).

Profiling studies of fusion genes in multifocal disease are also important to evaluate co-occurrence of these alterations. Other FISH analyses of recurrent ETS gene rearrangements in multifocal prostates showed complex patterns of alterations, with both rearranged and un-rearranged foci and multiple ETS rearrangements within the same gland (Clark et al., 2008). Moreover, these fusion events were found to be mostly mutually exclusive between foci and might represent effective clonal markers. However, exceptions were observed with multiple ETS rearrangements within the same tumor focus (Svensson et al., 2011). More recently, several investigations have shown that ETS gene fusion exclusivity or co-occurrence in prostate cancer is associated with several other factors and aberrations. Outlier expression of *SPINK1* had been reported in a subset of ETS-negative prostate cancer samples exclusively (Tomlins et al., 2008b). Later, ERG/SPINK1 immunohistochemistry analyses performed in different foci of prostate cancer samples revealed that *ERG* and *SPINK1* overexpression were mutually exclusive in all tumor foci (Fontugne et al., 2016). In another report, it was found that 17% of prostate cancer cases with multifocal tumors showed both *ERG* and *SPINK1* overexpression within different regions of either the same tumor focus or different foci, but not in the same tumor cells (Lu et al., 2020). Deletions in *CHD1* and *MAP3K7*, and mutations in *SPOP, FOXA1*, and *IDH1* were also found to be associated with the ETS-fusion negative subtype (Liu et al., 2007; Barbieri et al., 2012; Grasso et al., 2012; Cancer Genome Atlas Research Network, 2015). Interestingly the *SKIL* fusions described in Annala et al. (2015) and the RAF-kinase fusions described in Palanisamy et al. (2010) were identified in analyses performed on ETS rearrangements-negative cases. As many AR target genes are also regulated by ERG (Yu et al., 2010; Zhang et al., 2019), the ERG fusion positive cancers may have correlating expression of the androgen-driven fusion transcripts due to overexpression of *ERG*. For example, correlative analysis with other ETS gene fusions showed that KLK4-KLKP1 expression is associated with ERG but not ETV1, ETV4, or ETV5 (Chakravarthi et al., 2019). This may be explained by the presence of a strong ERG binding site at the fusion junction, suggesting that the expression of the KLK4-KLKP1 fusion gene is regulated by ERG in addition to AR. The diverse molecular heterogeneity within the ETS fusion-negative subtype, its clinical significance, and implication in designing novel therapeutic strategies has been recently reviewed in Bhatia and Ateeq (2019).

### Utility of Androgen-Driven Fusions in Prostate Cancer Diagnostics and Treatment

Tumor-specific gene fusions can serve as diagnostic biomarkers or help define molecular subtypes of tumors. For example, gene fusions involving ETS transcription factors have been utilized in diagnostic applications, such as with detection of TMPRSS2-ERG fusion transcripts in urine samples or CTCs from patients or ERG protein by immunostaining in biopsies [reviewed recently in Kumar-Sinha et al. (2015), Berg (2016), and Garcia-Perdomo et al. (2018)]. Despite the recently increased molecular understanding and array of prostate cancer molecular biomarkers available, molecular subtyping of prostate cancer with clinically relevant treatment stratification based on fusion genes and other genetic aberrations remains a challenge (Kohaar et al., 2019). In general, expression of the AR-driven fusions is inhibited along other AR targets by antiandrogens or androgen deprivation, but specific means to target the fusion products and their effects are rare (Bhatia and Ateeq, 2019; Pederzoli et al., 2020).

As the TMPRSS2-ERG fusion is the most common alteration in prostate cancer, molecular targeting of it has gained attraction as a potential therapeutic strategy. Recent examples include the work of Wang and colleagues, who identified a series of peptides that interact specifically with the DNA binding domain of ERG, leading to proteolytic degradation of the ERG protein, and attenuation of ERG-mediated transcription, chromatin recruitment, protein-protein interactions, cell invasion and proliferation, and tumor growth (Wang et al., 2017). Butler and colleagues identified and characterized a new class of small molecule ERG antagonists through rational *in silico* methods, demonstrating that a small molecule targeting the ERG-ETS domain suppressed its transcriptional activity and reverse transformed the characteristics of prostate cancers aberrantly expressing *ERG* (Butler et al., 2017). Treatment of prostate cancer cells with the USP9X inhibitor WP1130 resulted in ERG degradation both *in vivo* and *in vitro*, impaired the expression of genes enriched in *ERG* and prostate cancer relevant gene signatures, and inhibited growth of ERG-positive tumors in mouse xenograft models (Wang et al., 2014). Mohamed and colleagues screened small-molecule libraries for inhibition of ERG protein in TMPRSS2-ERG harboring VCaP cells and identified a small molecule that selectively inhibits erg-positive cancer cell growth (Mohamed et al., 2018).

On the basis of the interaction of ERG and other ETS fusions with the DNA repair proteins PARP1 and DNA-PKc, use of PARP inhibitors has shown initial promise and is being tested in ETS fusion-positive prostate cancers [reviewed in Kumar-Sinha et al. (2015) and Pederzoli et al. (2020)]. Going further downstream to find effective targets, characterization of the ERG-regulated kinome identified *TNIK* as a potential therapeutic target in ERG-fusion gene positive prostate cancer (Lee et al., 2019). Another small molecule inhibitor termed YK-4-279 was shown to be effective against ETV1 activity. In xenografts models YK-4-279 significantly reduced both primary tumor growth and metastasis to the lungs (Rahim et al., 2014).

While the therapeutic targeting of transcription factor oncogenes remains challenging, tumors with fusions involving therapeutically targetable genes, most often kinases, often have the strongest implications in personalized treatment of cancer patients. Amongst prostate cancer fusion genes, especially the effects of androgen-regulated SLC45A3-BRAF and a non-androgen-regulated ESRP1-RAF1 are targetable. The effects of ectopic expression of these fusion genes were studied in RWPE benign immortalized prostate epithelial cells and resulted in increased proliferation, invasion and anchorage-independent growth, which were sensitive to RAF and MEK inhibitors (Palanisamy et al., 2010). These results indicate that RAF-fusion-positive patients may respond to these drugs regardless of AR regulation of the fusion gene. Despite the low recurrence (1–2% in Caucasian, 4–6% in an Indian cohort (Ateeq et al., 2015), screening of these actionable RAF alterations could be beneficial in disease management of RAF-fusion-positive patients (Palanisamy et al., 2010; Bhatia and Ateeq, 2019; Pederzoli et al., 2020). Several FGFR inhibitors currently in clinical trials represent potential therapeutics for cancers harboring FGFR fusions [reviewed in Parker et al. (2014) and Krook et al. (2020)]. A rare interchromosomal fusion of *SLC45A3* with *FGFR2* in which the *SLC45A3* non-coding exon 1 is fused to the intact coding region of *FGFR2* has been found from a brain metastasis of a prostate cancer patient (Wu et al., 2013), indicating that there are also prostate cancer patients that likely benefit from these FGFR inhibitors. Further rare and potentially targetable, AR-driven fusions include for example PIK3C family gene fusion ACPP-PIK3CB and R-spondin fusion GRHL2-RSPO2 (Robinson et al., 2015).

Prostate cancer xenografts play a central role in pharmacological testing of potential drugs. The VCaP cell line, due to the TMPRSS2-ERG fusion gene it harbors, has been widely utilized in xenograft drug studies. For example, TMPRSS2-ERG harboring VCaP bone xenograft models were shown to better respond to enzalutamide treatment, suggesting that ERG expression status in tumors could help stratify patients for enzalutamide therapy (Semaan et al., 2019). TMPRSS2-ERG-targeted gene silencing therapy using liposomal nanovectors suppressed tumor growth in a VCaP xenograft model and enhanced the efficacy of docetaxel chemotherapy (Shao et al., 2020). While TMPRSS2-ERG activates NO-cGMP signaling in prostate cancer cells, sGC inhibitor treatment repressed tumor growth in TMPRSS2-ERG-positive VCaP xenograft models and acted in synergy with enzalutamide, the potent AR antagonist (Zhou et al., 2019). In the future, more of the specific marker-driven therapies are likely to be developed, especially through utilization of patient-derived 3D cultures as well as xenografts (PDXs) [recently reviewed in Kato et al. (2020), Palanisamy et al. (2020), and Risbridger et al. (2020)]. Patient-derived 3D cultures include spheroids and organoids, which are applicable in high throughput screening of e.g., drug libraries, while PDX models entail engrafting patient tissue in immunocompromised mice [reviewed in Kato et al. (2020) and Risbridger et al. (2020)]. Although an intact immune system against the tumor is missing from the PDXs, this experimental model retains many other valuable properties of tumor tissue and *in vivo* environment and is thus valuable in developing new drugs and selecting appropriate treatment strategies for prostate cancer patients. In terms of prostate cancer fusion genes, the expression of *ERG* has been shown to be retained in the PDXs along with other molecular, histopathologic, and genomic characteristics (Palanisamy et al., 2020), indicating PDXs to be a valuable strategy to assess fusion-specific therapeutic options in the future.

## Conclusions

The frequent gene fusions in prostate cancer are a curiosity amongst solid tumors. Why and how this particular tumor type benefits so much from these rearrangements for them to be so frequent are still open questions. While the benefit with certain fusions may clearly result from *de novo* expression of a cancer driver protein, for some fusions the advantage seems not as straightforwardly explained nor convincingly supported by functional data. Especially, despite a lot of effort, the field has yet to pinpoint why and how TMPRSS2-ERG fusion is an early event in prostate cancer development, yet the most significant functions of it seem concentrated in the phase of metastatic disease. The PCAWG Consortium recently reported that, amongst their 3,540 fusion events identified in 1,188 pan-cancer samples studied, 82% were associated with specific genomic rearrangements (PCAWG Transcriptome Core Group et al., 2020). For the remaining fusions, it is possible that the relevant genomic rearrangements have not been detected, or that fusions occur at the RNA level. Thus, up to a fifth of chimeric fusion transcript types may result from a trans-splicing or read-through event, which suggests that a significant number of non-genetic fusions are present also in prostate cancer. Furthermore, the existence of transcriptional read-through mechanisms suggests that, in addition to transcriptional deregulation, also splicing and RNA-binding regulatory mechanisms are functionally relevant for fusion transcript expression in prostate cancer.

The case of SLC45A3-ELK4 fusion has proven that it is possible for a chimeric RNA to function as a ncRNA, even though the 3′ fusion partner is initially protein-coding. Considering that chimeric transcripts may have acquired *de novo* structures and functions, it is possible that also some of the other fusion transcripts may have non-coding functions yet to be discovered. This is supported by the notion that up to 20% of expressed prostate cancer fusion transcripts are non-canonical, with one or both transcripts in antisense orientation (Vellichirammal et al., 2020). Furthermore, according to the data by Dehghannasiri and colleagues, up to 10% of prostate cancer fusions involve lncRNAs as the other partner (Dehghannasiri et al., 2019), making it likely that more AR-driven lncRNA fusions will be discovered. Thus, the fascinating field of prostate cancer fusions will presumably keep us entertained also in the foreseeable future.

## Author Contributions

MS and LL wrote the manuscript. SK designed the figures. MS, LL, and SK edited the manuscript. All authors have approved the final version of the manuscript.

## Conflict of Interest

The authors declare that the research was conducted in the absence of any commercial or financial relationships that could be construed as a potential conflict of interest.
